# Microbiological replacement associated with recurrent pneumonia during chemotherapy for Hodgkin lymphoma: a case report

**DOI:** 10.1099/acmi.0.001263.v3

**Published:** 2026-09-22

**Authors:** Kazuhiro Itoh, Nozomi Otsuki, Hiroshi Tsutani, Yasuhiko Mitsuke, Masamichi Ikawa, Hiromichi Iwasaki

**Affiliations:** 1 Department of Community Health Science, Faculty of Medical Sciences, University of Fukui, Fukui, Japan; 2 Department of Internal Medicine, NHO Awara National Hospital, Awara, Japan

**Keywords:** antimicrobial stewardship, chemotherapy, extended-spectrum beta-lactamase, hypermucoviscosity, *Klebsiella pneumoniae*, recurrent pneumonia

## Abstract

**Background.** Persistent or recurrent pneumonia during antimicrobial therapy in patients receiving chemotherapy can be difficult to interpret. It may reflect persistence of the initial pathogen, inadequate antimicrobial exposure, noninfectious mimics, aspiration, superinfection or acquisition of a new respiratory pathogen. We report a case illustrating the clinical importance of repeat microbiological assessment when pneumonia persists or worsens despite apparently appropriate antimicrobial treatment.

**Case presentation.** A man in his 80s with advanced classical Hodgkin lymphoma and chronic obstructive pulmonary disease developed pneumonia during chemotherapy. The initial sputum culture yielded cefepime-susceptible *Klebsiella oxytoca*. Cefepime produced transient defervescence, but oxygen requirements and radiographic abnormalities persisted, and fever recurred from day 18 with radiographic progression by day 19. Repeat sputum obtained on day 23 subsequently yielded extended-spectrum beta-lactamase-producing *Klebsiella pneumoniae* resistant to piperacillin/tazobactam and cefepime but susceptible to carbapenems. The isolate showed a positive string test, consistent with a hypermucoviscous phenotype. Doripenem was started after the resistant isolate was reported; however, the patient later developed progressive bilateral pulmonary infiltrates and respiratory failure and died. The interpretation of microbiological replacement remained presumptive because the organisms were recovered from expectorated sputum and molecular characterization was unavailable.

**Conclusion.** This case highlights presumptive microbiological replacement as a clinically important consideration when pneumonia persists or worsens during chemotherapy. Repeat respiratory culture and updated susceptibility testing can materially alter antimicrobial management. A positive string test should be interpreted as a supplementary microbiological clue rather than proof of hypervirulence, metastatic infection or causality for death.

**Impact Statement**

Persistent or recurrent pneumonia in immunocompromised hosts should prompt renewed microbiological assessment rather than an assumption that the initial pathogen remains responsible. In this case, repeat sputum culture revealed a different *Klebsiella* species with a markedly different susceptibility profile, changing antimicrobial management. Because both isolates were recovered from expectorated sputum and molecular characterization was unavailable, the microbiological replacement was presumptive. The case also emphasizes that hypermucoviscosity should not be treated as proof of hypervirulence or dissemination without supporting molecular or clinical evidence.

## Data Summary

No external datasets, code or software were generated or analysed for this case report. All data supporting the conclusions of this article are included within the article. Additional individual-level clinical data are not publicly available because they could compromise patient confidentiality.

## Introduction

Persistent or recurrent pneumonia during antimicrobial therapy in patients receiving chemotherapy is clinically challenging. Immunocompromised patients may have simultaneous infectious and noninfectious causes of pulmonary deterioration, and repeated microbiological assessment is often necessary when the clinical course changes [1, 2]. Persistence refers to continued evidence of the initial organism despite therapy, whereas relapse generally denotes recurrence after an initial response. Superinfection denotes a new infection emerging during treatment. In this report, we use the term ‘microbiological replacement’ descriptively to indicate recovery of a different bacterial species with a substantially different antimicrobial susceptibility profile during a persistent or worsening pulmonary course. This interpretation is presumptive and may clinically overlap with superinfection.

Extended-spectrum beta-lactamase (ESBL)-producing *Enterobacterales* are important causes of healthcare-associated infection and may require reassessment of empirical beta-lactam therapy [3]. Hypermucoviscosity, often detected by a positive string test, is a phenotypic feature of some *Klebsiella pneumoniae* isolates, but it should not be equated with hypervirulence without molecular and clinical evidence [4, 5]. This case describes a presumptive microbiological shift from *Klebsiella oxytoca* to ESBL-producing *K. pneumoniae* during persistent pneumonia in a patient receiving chemotherapy, emphasizing repeat culture, antimicrobial reassessment and cautious interpretation of microbiological phenotypes.

## Case presentation

On day 1, a man in his 80s with classical Hodgkin lymphoma, lymphocyte-depleted variant, stage IVB, was admitted with high-grade fever of 39 °C and hypoxaemia, with oxygen saturation of 90% on room air, after five cycles of brentuximab vedotin, doxorubicin, vinblastine and dacarbazine. His medical history included hypertension and chronic obstructive pulmonary disease (COPD). Baseline spirometric data, including FEV1, were unavailable; he did not use long-term home oxygen. He had received no systemic antibacterial therapy immediately before admission. Computed tomography of the chest on admission showed right-sided pulmonary infiltrates and bilateral pleural effusions.

At the initial episode, the white blood cell count was 8.1×10⁹ l^−1^, with neutrophils 63.3% (absolute neutrophil count ~5.1×10⁹ l^−1^) and lymphocytes 35.8% (absolute lymphocyte count ~2.9×10⁹ l^−1^); haemoglobin was 9.7 g dl^−1^ and the platelet count was 213×10⁹ l^−1^. C-reactive protein was 5.42 mg dl^−1^. Procalcitonin was not measured at the initial episode. Beta-d-glucan was 32 pg ml^−1^. Renal function was preserved, with creatinine 0.82 mg dl^−1^, blood urea nitrogen 24.5 mg dl^−1^ and estimated glomerular filtration rate 67.4 ml min^−1^/1.73 m². Blood lactate was 7 mg dl^−1^ (~0.8 mmol l^−1^). Blood pressure was 94/60 mmHg, pulse rate was 118 beats/min and respiratory rate was 30 breaths/min. Arterial blood gas analysis on day 1 yielded a PaO_2_/FiO_2_ ratio of 145. The maximum oxygen requirement during the initial episode was 5 l min^−1^. The pneumonia severity was classified as age, dehydration, respiratory failure, orientation disturbance and low blood pressure (A-DROP) score 3, based on age, dehydration and oxygen desaturation.

## Investigations and microbiological findings

During the initial episode, sputum and blood cultures were obtained. Sputum culture yielded *K. oxytoca*, whereas blood cultures were negative. The initial sputum specimen was Geckler group 1 [6]. Susceptibility testing showed susceptibility to cefepime, piperacillin/tazobactam, ceftriaxone, ceftazidime and carbapenems, and the reported profile did not support ESBL production. Cefepime 2 g twice daily was started. Defervescence was transient, while oxygen requirements persisted and respiratory status remained unstable.

**Fig. 1.**
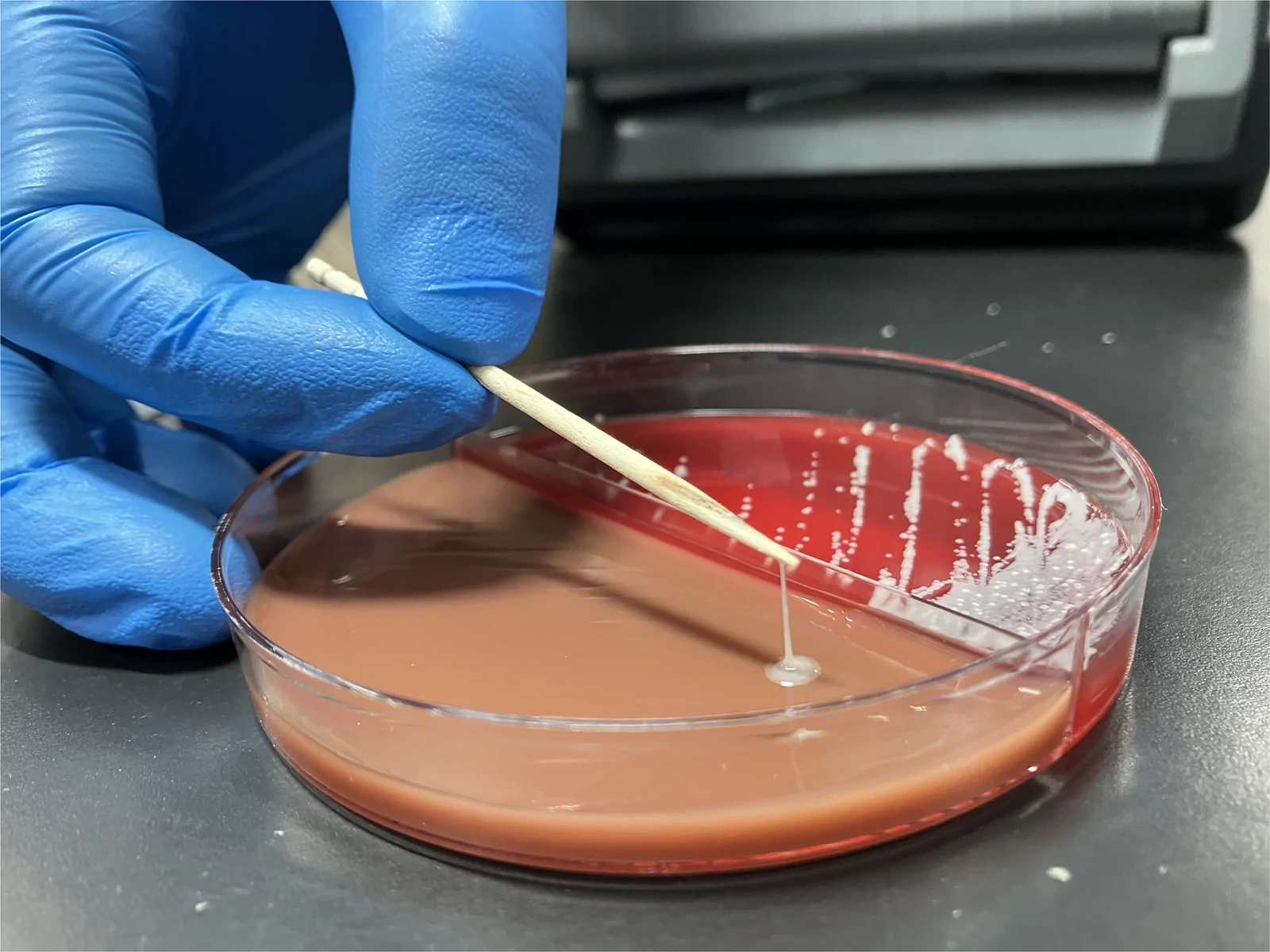
Positive string test of the *K. pneumoniae* isolate showing the formation of a viscous filament greater than 5 mm, consistent with a hypermucoviscous phenotype.

Fever recurred from day 18 onward. Chest radiography on day 19 showed worsening of the right-sided infiltrate and a new infiltrate in the left lower lung field. On day 23, computed tomography showed further worsening of the right-sided infiltrate, bilateral lower-lobe atelectasis and increased bilateral pleural effusions. The A-DROP score was 3, based on age, oxygen desaturation and impaired consciousness [Glasgow Coma Scale (GCS) score 10], without dehydration. Repeat sputum obtained on day 23 subsequently yielded *K. pneumoniae* rather than *K. oxytoca*. The repeat sputum specimen was Geckler group 3 [6]. The colonies were markedly mucoid, and the isolate showed a positive string test with formation of a viscous filament greater than 5 mm (Fig. 1). Whole-body contrast-enhanced computed tomography revealed no liver abscess or other deep abscess formation.

Blood cultures were not repeated during the worsening pulmonary course. The treating team focused repeat microbiological evaluation on sputum because the deterioration was interpreted as persistent pulmonary infection despite cefepime; the absence of repeat blood cultures is acknowledged as a limitation. Respiratory viral testing was not performed. Urine culture yielded *Corynebacterium* species in the absence of pyuria and was not considered supportive of urinary tract infection. Pleural fluid was not sampled. Beta-d-glucan was 38 pg ml^−1^ on day 25; Aspergillus antigen, cryptococcal antigen and Candida mannan antigen were negative. *Pneumocystis jirovecii* PCR was not performed, and the patient had not received *Pneumocystis* prophylaxis. *Pneumocystis* pneumonia was considered less likely because beta-d-glucan showed little interval change and computed tomography did not show a typical diffuse ground-glass pattern, but it could not be excluded. Bronchoscopy with bronchoalveolar lavage was considered but could not be performed because bronchoscopy was not available at the treating institution. Procalcitonin on day 23 was <0.20 ng ml^−1^.

Both *Klebsiella* isolates were identified by MALDI-TOF MS using the BD Bruker MALDI Biotyper sirius system (Becton Dickinson Japan, Tokyo, Japan) with the Bruker MALDI Biotyper Library BDAL version 13. Antimicrobial susceptibility testing was performed by an external reference laboratory. The *K. pneumoniae* isolate was reported by that laboratory as ESBL-producing; however, the exact confirmatory method used to establish the ESBL phenotype in this individual isolate could not be ascertained retrospectively. The isolate was reported as resistant to piperacillin/tazobactam, ceftriaxone, ceftazidime and cefepime but susceptible to carbapenems. Representative MICs included piperacillin/tazobactam >64 µg ml^−1^, ceftriaxone >32 µg ml^−1^, ceftazidime 32 µg ml^−1^, cefepime >16 µg ml^−1^, imipenem/cilastatin ≤1 µg ml^−1^ and meropenem ≤0.12 µg ml^−1^. Table 1 summarizes the susceptibility profiles of the two isolates.

**Table 1.** Antimicrobial susceptibility profiles of the initial *K. oxytoca* isolate and the subsequent ESBL-producing *K. pneumoniae* isolate

Antimicrobial agent	*K. oxytoca* MIC (interpretation)	*K. pneumoniae* MIC or category (interpretation)
Ampicillin (ABPC)	>16 (R)	>16 (R)
Sulbactam/ampicillin (SBT/ABPC)	≤8 (S)	>16 (R)
Piperacillin (PIPC)	≤8 (S)	>64 (R)
**Piperacillin/tazobactam (TAZ/PIPC**)	**≤8 (S**)	**>64 (R**)
Cefazolin (CEZ)	>4 (R)	>4 (R)
Cefotiam (CTM)	≤8 (S)	>16 (R)
Cefmetazole (CMZ)	≤16 (S)	≤16 (S)
Ceftriaxone (CTRX)	≤1 (S)	>32 (R)
Cefpodoxime proxetil (CFPN-PI)	≤1 (S)	>2 (R)
Ceftazidime (CAZ)	≤4 (S)	32 (R)
Sulbactam/cefoperazone (SBT/CPZ)	≤16 (S)	>32 (R)
**Cefepime (CFPM**)	**≤2 (S**)	**>16 (R**)
Cefozopran (CZOP)	Category only (S)	Category only (R)
**Doripenem (DRPM**)	**Category only (S**)	**Category only (S**)
**Imipenem/cilastatin (IPM/CS**)	**≤1 (S**)	**≤1 (S**)
**Meropenem (MEPM**)	**≤0.12 (S**)	**≤0.12 (S**)
Gentamicin (GM)	≤2 (S)	≤2 (S)
Amikacin (AMK)	≤4 (S)	≤4 (S)
Minocycline (MINO)	≤4 (S)	8 (I)
Ciprofloxacin (CPFX)	≤0.06 (S)	≤0.06 (S)
Levofloxacin (LVFX)	≤0.12 (S)	≤0.12 (S)
Trimethoprim/sulfamethoxazole (ST)	≤40 (S)	≤40 (S)

For doripenem and cefozopran, only categorical susceptibility results were available from the laboratory reports, and MIC values were not provided. Bold type highlights agents central to treatment decisions: transition from piperacillin/tazobactam and cefepime susceptibility to resistance, with retained carbapenem susceptibility.

I, intermediate; R, resistant; S, susceptible.

## Differential diagnosis

During the worsening pulmonary course, the main diagnostic considerations were persistence of the initial *K. oxytoca* pneumonia, hospital-acquired superinfection or presumptive microbiological replacement with a different bacterial pathogen, aspiration, non-bacterial infection, tumour fever, drug-related fever, chemotherapy-related pulmonary toxicity and lymphoma-related pulmonary involvement. Swallowing dysfunction was present and therefore aspiration remained plausible; vomiting occurred only on day 39 and did not explain the earlier radiographic progression. *Pneumocystis* pneumonia was considered less likely for the reasons noted above but was not definitively excluded. The chemotherapy regimen did not contain bleomycin; nevertheless, pulmonary toxicity has rarely been reported with brentuximab vedotin [7]. The focal and stepwise radiographic progression accompanied by recovery of different bacterial species supported an infectious process, although drug-related pneumonitis could not be excluded.

Several host-related and noninfectious factors could also have contributed to respiratory failure. The patient had advanced stage IV classical Hodgkin lymphoma, COPD and recent exposure to combination chemotherapy, all of which could have reduced pulmonary reserve. Both bacterial isolates were recovered from expectorated sputum rather than a sterile site, and the Geckler classifications indicated suboptimal specimen quality; therefore, colonization or upper-airway contamination cannot be excluded. Nevertheless, the change in recovered species and susceptibility profile occurred during persistent fever and progressive radiographic abnormalities and was considered clinically relevant without establishing causality.

## Treatment

Cefepime 2 g twice daily was continued from day 1 through day 23 because defervescence was initially obtained despite persistent oxygen requirements and an unstable respiratory course. With recurrent fever and radiographic progression, hospital-acquired and aspiration pneumonia were considered, and treatment was changed empirically on day 23 to piperacillin/tazobactam 4.5 g three times daily. At that point, there was no microbiological evidence of ESBL production, and piperacillin/tazobactam was selected as a carbapenem-sparing empirical regimen. Repeat culture and susceptibility testing were outsourced to an external reference laboratory, contributing to a reporting interval of ~7 days; the detailed internal laboratory workflow could not be reconstructed. After the repeat isolate was reported as ESBL-producing *K. pneumoniae* resistant to piperacillin/tazobactam and cefepime, doripenem 0.25 g three times daily was started on day 31. Renal function at doripenem initiation was preserved, with creatinine 0.54 mg dl^−1^ and estimated glomerular filtration rate 106.4 ml min^−1^/1.73 m².

Doripenem was selected for the reported ESBL-producing *K. pneumoniae* pneumonia based on the susceptibility profile. The regimen of 0.25 g three times daily was within the approved standard adult dosing range in Japan and was not a renal dose reduction; however, higher approved dosing can be considered for severe or refractory infection. Possible central nervous system infection was considered only after terminal neurological deterioration, after doripenem had already been selected for pneumonia. Cerebrospinal fluid examination could not be performed, and meningitis-directed optimization of antimicrobial therapy was not undertaken during the terminal deterioration.

## Outcome and follow-up

**Fig. 2.**
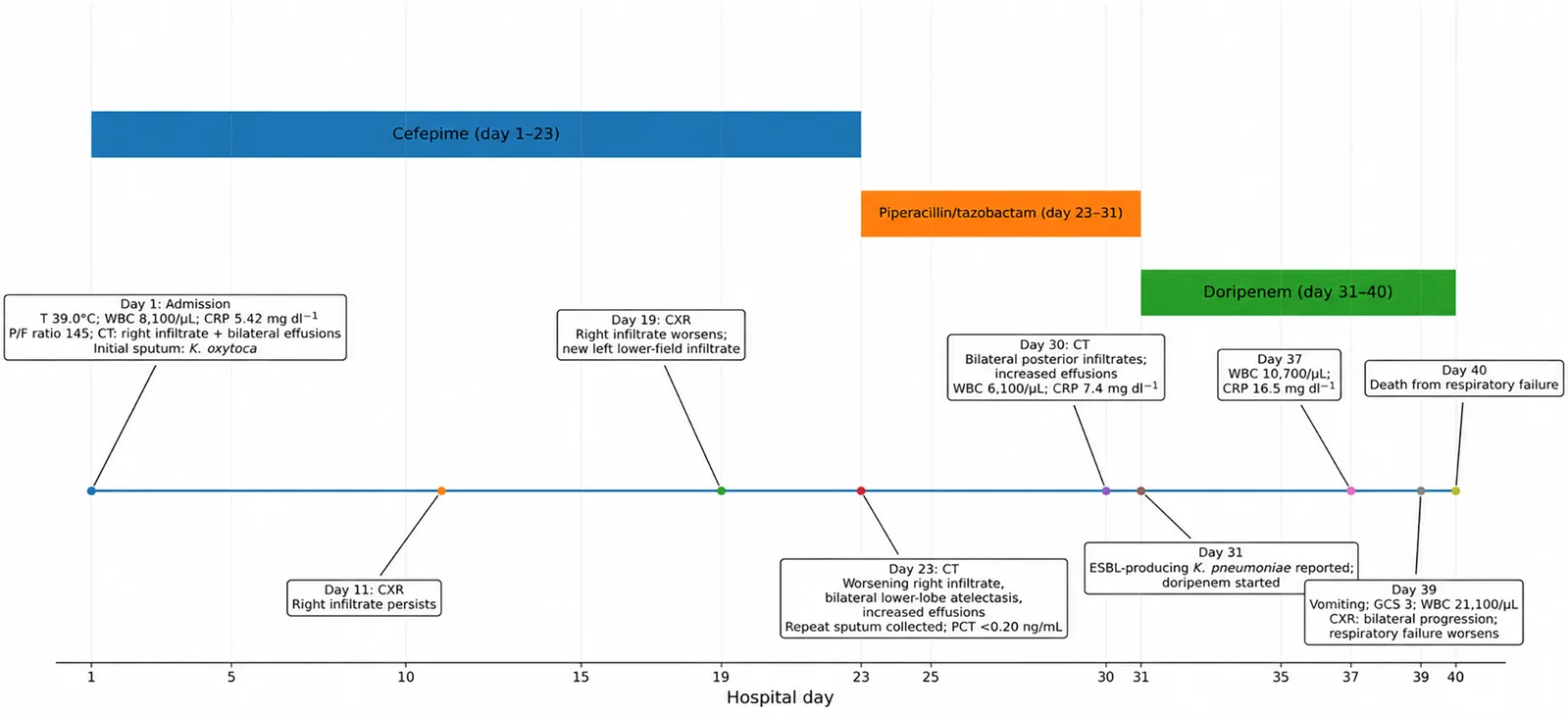
Clinical timeline showing antimicrobial therapy, major radiological and microbiological events, selected inflammatory markers and outcome. P/F, ratio of arterial oxygen partial pressure to fraction of inspired oxygen; PCT, procalcitonin.

On day 39, 8 days after doripenem initiation, the patient developed vomiting, profound impaired consciousness (GCS score 3) and increased work of breathing. White blood cell count had risen to 21.1×10⁹ l^−1^; procalcitonin was not repeated. Oxygen saturation decreased to 80% despite 7 l min^−1^ oxygen by mask, and oxygen was increased to 10 l min^−1^ via a reservoir mask, the maximum oxygen delivery used at the institution. Chest radiography showed expansion of bilateral pulmonary infiltrates and pleural effusions. Non-invasive ventilation and invasive mechanical ventilation were not instituted, and invasive ventilation was not pursued in accordance with the patient’s wishes and documented do-not-attempt-resuscitation status. The patient died of respiratory failure on day 40, ~1 month after the initial onset of pneumonia. The overall clinical course is summarized in Fig. 2.

## Discussion

This case is best interpreted as persistent pneumonia with presumptive microbiological replacement or superinfection rather than as a proven fatal disseminated infection due to ESBL-producing *K. pneumoniae*. The initial *K. oxytoca* isolate was susceptible to cefepime and piperacillin/tazobactam, whereas the subsequent *K. pneumoniae* isolate was reported as ESBL-producing and resistant to both agents. Because both organisms were recovered from expectorated sputum of suboptimal quality, microbiological replacement cannot be proven. Nevertheless, recovery of a different species with a markedly different susceptibility profile during radiographic progression changed antimicrobial management.

The temporal sequence is compatible with more than one mechanism. Prolonged cefepime exposure may have exerted antimicrobial selection pressure on colonizing flora, whereas acquisition of a new healthcare-associated organism followed by colonization or aspiration is also plausible. Because the isolates were different species and stored isolates were unavailable, the route of acquisition and the relationship between the organisms could not be further characterized.

From an antimicrobial stewardship perspective, persistent or worsening pneumonia after an initial response should prompt renewed sampling and reassessment of antimicrobial therapy rather than automatic continuation of therapy directed at the first isolate or indiscriminate broadening. In this case, piperacillin/tazobactam was selected before the ESBL phenotype was known to preserve carbapenem activity, whereas escalation to a carbapenem became microbiologically justified after the resistant *K. pneumoniae* isolate was reported [3]. The case cannot determine whether earlier empirical carbapenem therapy would have altered the outcome.

The second *K. pneumoniae* isolate showed a positive string test, indicating a hypermucoviscous phenotype. However, hypermucoviscosity and hypervirulence are not synonymous, and string-test positivity alone cannot define a hypervirulent strain [4, 5]. ESBL-producing hypermucoviscous *K. pneumoniae* has been reported in Japan [8, 9], and one comparative study found higher resistance to cefepime and piperacillin/tazobactam among hypermucoviscous ESBL-producing isolates [8]. In the present case, molecular virulence testing was not performed, blood cultures were not repeated during deterioration and evaluation for metastatic complications was incomplete, although contrast-enhanced computed tomography showed no liver or other deep abscess. Accordingly, the string test should be regarded as a phenotypic clue rather than evidence of hypervirulence, dissemination or causality for death.

Antimicrobial management also requires cautious interpretation. Cefepime was active against the initial isolate *in vitro*, but the prolonged course coincided with incomplete respiratory improvement and may have contributed to selection pressure. Piperacillin/tazobactam was used empirically before the second susceptibility profile was available; once resistance to piperacillin/tazobactam and cefepime was reported, transition to a carbapenem was consistent with contemporary guidance for serious ESBL-producing *Enterobacterales* infections [3]. Doripenem 0.25 g three times daily was within the approved Japanese adult dosing range, although a higher dose could have been considered for severe or refractory infection. Because the susceptibility result became available only after an ~7-day external-laboratory turnaround and deterioration continued despite subsequent doripenem, this case cannot determine whether earlier escalation, a higher doripenem dose or a different carbapenem regimen would have changed the outcome.

This report has important limitations. Blood cultures were not repeated during clinical deterioration, and bloodstream dissemination was not demonstrated. Respiratory viral testing, *Pneumocystis* PCR and bronchoalveolar lavage were not performed; bronchoscopy was unavailable at the treating institution. Both sputum specimens were of suboptimal quality (Geckler groups 1 and 3), so colonization or upper-airway contamination cannot be excluded. The exact confirmatory method used by the external reference laboratory to establish the ESBL phenotype could not be verified retrospectively. The isolates were no longer available for molecular characterization of ESBL genes, virulence determinants or strain typing. Central nervous system involvement was suspected only terminally and was not confirmed. Baseline FEV1 and serial arterial blood gas measurements were unavailable, and procalcitonin was not repeated on day 39. Important noninfectious contributors to respiratory failure, including advanced lymphoma, COPD, aspiration and chemotherapy-related pulmonary toxicity, could not be fully excluded. These limitations argue against attributing the fatal outcome solely to the reported ESBL-producing *K. pneumoniae* isolate.

## Conclusion

In patients receiving chemotherapy, persistent or worsening pneumonia may be associated with emergence or acquisition of a different resistant organism rather than persistence of the initially recovered pathogen. Repeat microbiological assessment and updated susceptibility testing can materially alter antimicrobial management. Hypermucoviscosity should be interpreted cautiously when molecular characterization and evaluation for invasive disease are incomplete.

## Abbreviations

A-DROP: age, dehydration, respiratory failure, orientation disturbance and low blood pressure
COPD: chronic obstructive pulmonary disease
ESBL: extended-spectrum beta-lactamase
GCS: Glasgow Coma Scale

## References

[1] Ramirez JA, Musher DM, Evans SE, Dela Cruz C, Crothers KA (2020). Treatment of community-acquired pneumonia in immunocompromised adults: a consensus statement regarding initial strategies. Chest.

[2] Azoulay E, Russell L, Van de Louw A, Metaxa V, Bauer P (2020). Diagnosis of severe respiratory infections in immunocompromised patients. Intensive Care Med.

[3] Tamma PD, Heil EL, Justo JA, Mathers AJ, Satlin MJ (2024). Infectious diseases society of America 2024 guidance on the treatment of antimicrobial-resistant gram-negative infections. Clin Infect Dis.

[4] Russo TA, Marr CM (2019). Hypervirulent *Klebsiella pneumoniae*. Clin Microbiol Rev.

[5] Catalán-Nájera JC, Garza-Ramos U, Barrios-Camacho H (2017). Hypervirulence and hypermucoviscosity: Two different but complementary *Klebsiella* spp. phenotypes?. Virulence.

[6] Geckler RW, Gremillion DH, McAllister CK, Ellenbogen C (1977). Microscopic and bacteriological comparison of paired sputa and transtracheal aspirates. J Clin Microbiol.

[7] Khalil ORS, Mallah SMA, Owda F, Salim H, Mallah H (2024). Brentuximab-induced pneumonitis and organizing pneumonia: a case report with literiture review. Ann Med Surg (Lond).

[8] Tanimoto H, Shigemura K, Osawa K, Kado M, Onishi R (2023). Comparative genetic analysis of the antimicrobial susceptibilities and virulence of hypermucoviscous and non-hypermucoviscous ESBL-producing *Klebsiella pneumoniae* in Japan. J Microbiol Immunol Infect.

[9] Ukai K, Tomoda Y, Ishii S, Nakazato S, Doi Y (2025). A case of fatal disseminated infection with masticator space abscess caused by ESBL-producing K57-ST412 hypervirulent *Klebsiella pneumoniae*. J Infect Chemother.

